# Rapid Characterization and Action Mechanism of the Antidiabetic Effect of *Diospyros lotus* L Using UHPLC-Q-Exactive Orbitrap MS and Network Pharmacology

**DOI:** 10.1155/2022/8000126

**Published:** 2022-12-31

**Authors:** Shihan Qin, Mingjuan Liu, Sunv Tang, E. Shuai, Ziming Wang, Kaiquan Yu, Wei Cai

**Affiliations:** ^1^School of Pharmaceutical Sciences, Sino-Pakistan Center on Traditional Chinese Medicine, Hunan University of Medicine, Huaihua 418000, China; ^2^School of Pharmacy, Weifang Medical University, Weifang 261000, China

## Abstract

*Diospyros lotus* L, F. Ebenaceae, is an edible fruit that is widely distributed in China and other Asian countries. Presently, *Diospyros lotus* L can be used to treat patients with diabetes; however, its chemical composition and pharmacological profiles remain to be elucidated. This study investigated the potential bioactive compounds of *Diospyros lotus* L and their mechanisms of action using LC-MS and network pharmacology analysis. First, the components of *Diospyros lotus* L were identify using a reliable strategy for UHPLC-Q-Exactive Orbitrap mass spectrometry combined with parallel reaction monitoring (PRM) in the negative ion mode. Second, a network pharmacology study, including target gene prediction and functional enrichment, was applied to screen the main quality markers of *Diospyros lotus* L and explore its potential mechanism for the treatment of diabetes. The results showed that a total of 159 compounds were identified from *Diospyros lotus* L, among which, 140 were reported for the first time. Furthermore, 40 active components, such as quercetin, luteolin, and kaempferol, were proposed as active components of *Diospyros lotus* L for the treatment of diabetes based on network pharmacology analysis. In addition, 92 relevant antidiabetic targets were mainly related to positive regulation of transcription from the RNA polymerase II promoter, extracellular space, and protein binding, suggesting the involvement of TNF, PI3K-Akt, and HIF-1 signaling pathways in the antidiabetic effect of *Diospyros lotus* L. Our results may provide a useful approach to identify potential active components and molecular mechanisms of *Diospyros lotus* L for the treatment of diabetes.

## 1. Introduction

*Diospyros lotus* L, a genus of the family Ebenaceae, is an edible fruit that is widely distributed in China and other Asian countries. *Diospyros lotus* L fruit extract has antidiabetic, antitumor, antinociceptive, and anti-inflammatory effects [1–4] and is used for treating various diseases, such as hypertension, diarrhea, and dry cough. However, to date, few studies have investigated the chemical composition and mechanism of the antidiabetic effect of *Diospyros lotus* L.

Diabetes is a chronic, progressive, and complex metabolic disease characterized by hyperglycemia, which is caused by insufficient insulin secretion, insufficient function, or the simultaneous occurrence of both [5, 6]. Owing to the long-term effects of hyperglycemia, various diabetic complications can occur. These complications not only cause great harm to the physiological and psychological status of the patients but also put enormous pressure on society [7].

Extracts of *Diospyros lotus* L and its compounds have hypoglycemic effects [8–10]. However, the pharmacological mechanisms and bioactive components of *Diospyros lotus* L remain unknown. Network pharmacology is based on the chemical components of traditional Chinese medicine in the existing database to explore its mechanism from multiple perspectives, such as target gene identification and function prediction [11, 12].

In this study, a UHPLC-Orbitrap-MS combined with PRM was developed for component identification of *Diospyros lotus* L. The bioactive ingredients and mechanism of action of *Diospyros lotus* L on the targets of diabetes were investigated by network pharmacology, which is of great significance for further research on *Diospyros lotus* L.

## 2. Methodology

### 2.1. Materials and Chemicals

HPLC-grade acetonitrile and methanol were obtained from Merck Company Inc. (Darmstadt, Germany), and formic acid was obtained from Fisher Chemicals (Fairlawn, NJ, USA). Purified water was purchased from the A.S. Watson Group Ltd. (Hong Kong). Other reagents and chemicals were of analytical grade and were supplied by the Aladdin Industrial Corporation. Dried *Diospyros lotus* L samples were collected from Shexian County, Hebei Province, China, in November of each year.

Reference standards, including neochlorogenic acid, chlorogenic acid, 1,3-dicaffeoylquinic acid, isochlorogenic acid A, isochlorogenic acid B, and isochlorogenic acid C, were obtained from Chengdu Herbpurify Co., Ltd. Procyanidin, phlorizin, trilobatin, and phloretin were acquired from Sichuan Weikeqi Biological Technology Co., Ltd. Quinic acid, ferulic acid, catechin, quercetin, quercitrin, quercetin 3-O-rutinoside, myricitrin, isoquercitrin, nicotiflorin, myricetin, eriodictyol, luteolin, naringenin, and kaempferol were purchased from Chengdu Purechem Standard Co., Ltd. The purity of all standard compounds was no less than 98% based on LC-UV.

### 2.2. Standard and Sample Preparation

*Diospyros lotus* L (1 g) was extracted with 70% methanol (20 mL) by sonication (1 h). Then, the extract was centrifuged (15 min, 10°C, 12000 rpm) to obtain the supernatant. Finally, 2 *μ*L of the supernatant was injected into the LC-MS system for analysis.

All the reference standards were accurately weighed using an electronic analytical balance (1 mg) and dissolved in methanol (1 mL). Then, 10 *μ*L of each standard solution was added to a 1-mL volumetric flask to prepare a mixed standard solution. The obtained standard solution was stored below 4°C before analysis.

### 2.3. Chromatography and MS Conditions

Chromatographic separation was performed on a Dionex Ultimate 3000 UHPLC (Thermo Fisher Scientific, San Jose, CA, USA), using a Thermo Scientific Hypersil GOLDTM aQ (100 mm × 2.1 mm, 1.9 *μ*m).

The column compartment was maintained at 40°C, and the flow rate was set at 0.3 mL/min. Water containing 0.1% formic acid (solvent system A) and acetonitrile (solvent system B) served as the mobile phase. The gradient elution program was as follows: 0–2 min, 95%–5% B; 2–5 min, 80%–20% B; 5–10 min, 75%–25% B; 10–12 min, 45%–5% B; 12–20 min, 20%–80% B; 20–25 min, 5%–95% B; 25–30 min, 95%–5% B.

Mass detection was performed on a Q-Exactive Orbitrap MS equipped with an electrospray ionization source operating in negative mode with the following operating parameters: spray voltage at −3.0 kV; sheath gas flow rate at 30 arbs; auxiliary gas flow rate at 10 arbs; capillary temperature at 320°C; heater temperature at 350°C; S-lens RF level at 50; and normalized collision energies at 30%. The MS spectra were recorded over an *m/z* range of 80–1000. All data were acquired and processed using Xcalibur software version 4.2.

### 2.4. Candidate Ingredient Screening

To select the components that have better biological availability in vivo, the components were filtered using the principle of “drug-like soft” in FAFDrugs4. Screening parameters included restriction to molecular weight, logP, and hydrogen bond acceptors (HBA). Details of the physicochemical property filters are listed in Table 1.

### 2.5. Targets of *Diospyros lotus* L and Diseases

The targets of the filtered components were obtained from the traditional Chinese medicine systems pharmacology database and analysis Platform (TCMSP) and predicted using Swiss TargetPrediction (STP). Setting the organism “Homo sapiens” and targets with a probability value greater than 0.1 were considered as potential effective targets for these compounds in the STP database.

Diabetes-related targets were searched in the Online Mendelian Inheritance in Man and GeneCards platform with “diabetes” as the keyword. The collected targets were amalgamated and duplicated. Potential target genes of *Diospyros lotus* L therapy for diabetes were obtained through the jvenn intersection.

### 2.6. Construction of Protein-Protein Interaction (PPI) Network

The PPI network between target proteins of the related ingredients in *Diospyros lotus* L and diabetes was obtained by STRING and then imported into Cytoscape software version 3.8.2 to construct and validate a visual network. The species was set as “Homo sapiens,” and the protein interaction was obtained with a medium confidence score of 0.4 to ensure the reliability of our analysis. In the PPI network, topology parameters were calculated to obtain promising candidate targets that were visually characterized by the colors of nodes and to screen remarkable targets.

### 2.7. Enrichment Analysis

Gene Ontology (GO) and KEGG pathway enrichment analyses were performed using DAVID software. Subsequently, correlated “histograms” and “bubble graphs” were established.

### 2.8. Construction of Active Component-KeyGene-Pathway Interaction Network

To further explore the mechanism of the antidiabetic effect of *Diospyros lotus* L, an active component-keygene-pathway interaction network was constructed using Cytoscape 3.9.0 software. In the network, nodes with different shapes represented the active compounds, key genes, and related pathways, and an “edge” was an association between the nodes.

## 3. Results and Discussion

### 3.1. Establishment of Qualitative Analysis Strategy

In this study, an analytical method of UHPLC-Q-Exactive Orbitrap MS combined with the acquisition mode of the PRM mode was used to identify the chemical components of *Diospyros lotus* L. First, the extraction method and UHPLC-MS conditions of *Diospyros lotus* L were optimized. Second, the sample was injected into the UHPLC-Q-Exactive Orbitrap MS to obtain high-resolution mass data, including MS and MS^2^. Third, the compounds were predicted using the Compound Discover version 3.0 workstation with the aid of the metabolism workflow template by adjusting relevant parameters. Finally, the compounds were characterized based on full-scan MS and MS^2^, retention times, standards, and literature.

### 3.2. Optimization of the Extraction Method

To obtain the maximum extraction yield, the extraction method for *Diospyros lotus* L was optimized with respect to time (0.5, 1, and 2 h); solvent type (methanol and ethanol); solvent concentration (60%, 70%, and 80%); and liquid-to-solid ratio (10 : 1, 15 : 1, and 20 : 1). The optimal extraction method was ultrasonic extraction with 70% methanol (20 ml) for 1 h.

### 3.3. Optimization of UHPLC-MS Conditions

To achieve good chromatographic separation, UHPLC parameters were optimized, including the mobile phase (methanol/water and acetonitrile/water); type and content of acid (acetic acid and formic acid, 0.05%, 0.1%, and 0.2%); column (Waters ACQUITY BEH C18 column, 100 mm × 2.1 mm, 1.7 *μ*m, and HYPERSIL GOLD C18 column, 100 mm × 2.1 mm, 1.9 µm); column temperature (30, 35, and 40°C); flow rate of the mobile phase (0.2, 0.3, and 0.4 mL/min); and the difference gradient of mobile phase.. The MS parameters, including the flow rate of the sheath gas and auxiliary, temperature of the capillary and auxiliary, heater temperature, spray voltage, and collision energies were examined. In the optimized conditions of UHPLC-Q-Exactive Orbitrap MS, most of the components in the *Diospyros lotus* L showed efficient separation and parent/daughter ion pairs with high responses.

### 3.4. Characterization of *Diospyros lotus* L

**Figure 1:**
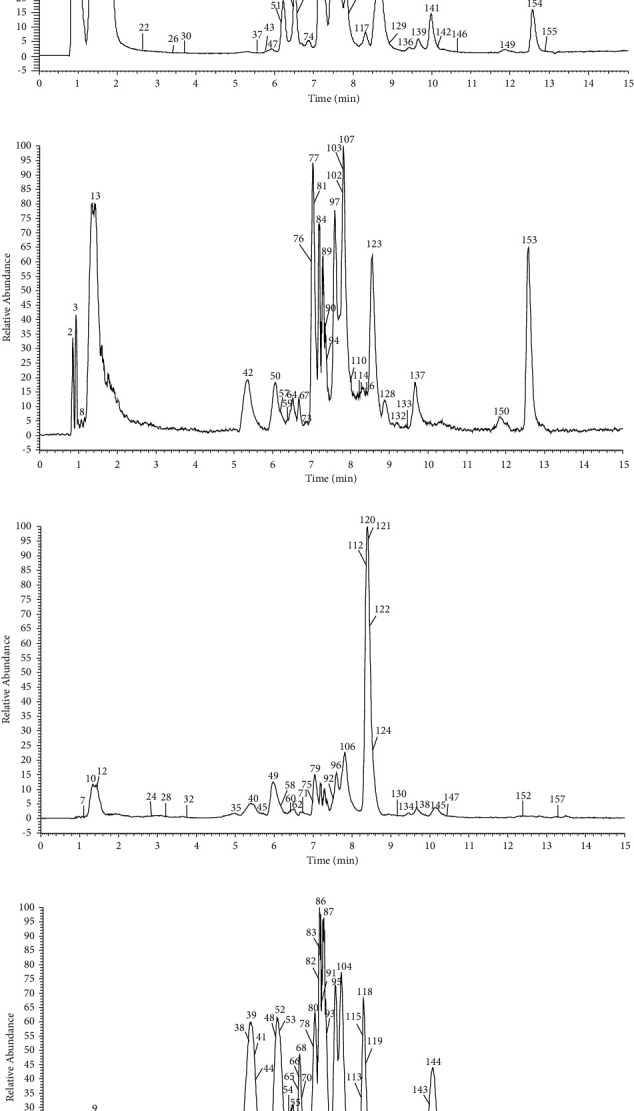
The high-resolution extraction ion chromatography of *Diospyros lotus* L in negative ion mode (a) 167.0350, 169.0142, 191.0561, 193.0350, 218.1030, 271.0612, 287.0561, 331.0671, 355.1035, 423.0416, 431.0983, 447.0933, 463.0882, 479.0831, 597.1825, 641.1359, 755.2040; (b) 151.0401, 167.0350, 177.0193, 191.0561, 271.0612, 287.0561, 303.0510, 319.0459, 331.0671, 423.0417, 433.1140, 435.1297, 463.0882, 465.1038, 477.1038, 507.1144, 579.2083, 609.146, 613.1779, 625.1410, 641.1359, 755.2040; (c) 145.0506, 151.0401, 153.0193, 167.0350, 177.01933, 179.0350, 187.0974, 193.0506, 263.1283, 273.0768, 285.0405, 287.0561, 299.0772, 303.0510, 315.0505, 317.0303, 319.0460, 329.0878, 331.0671, 353.0878, 433.11402, 435.1297, 449.1089, 457.1351, 461.0725, 463.0890, 465.1038, 475.1457, 477.0675, 491.1195, 493.0624, 507.1144, 515.1195, 579.1719, 579.2083, 609.1461, 613.1779, 615.0992, 625.1410, 641.1359; (d) 145.0506, 151.0401, 153.0193, 167.0350, 177.0193, 179.0350, 183.0299, 193.0506, 263.1283, 281.1396, 301.0354, 303.0510, 315.0722, 319.0459, 353.0878, 359.0984, 433.1140, 435.1297, 449.1089, 457.1351, 475.1457, 477.0675, 477.1038, 491.1195, 493.0624, 493.1199, 505.0987, 579.2083, 593.1512, 609.1461, 613.1779, 615.0992, 771.1989.

A total of 159 compounds, including 88 flavonoids, 24 phenylpropanoids, and 47 organic acids, were tentatively identified by UHPLC-Q-Exactive Orbitrap mass spectrometry; among them, 140 were reported for the first time in *Diospyros lotus* L. The chromatographic and mass data for the detected constituents are presented in Table 2. The extracted ion chromatogram in negative ion mode is shown in Figure 1.

#### 3.4.1. Identification of the Flavonoids in *Diospyros lotus* L

Compounds 36, 97, 98, 107, 117, 125, 128, 134, 144, 147, 150, 151, 153, 155, 157, and 159 were found at 4.99, 7.60, 7.60, 7.82, 8.33, 8.67, 8.93, 9.36, 9.45, 10.13, 10.33, 11.90, 12.11, 12.58, 12.82, 13.19, and 14.46 min, respectively. They were accurately identified as catechin, quercetin 3-O-rutinoside, isoquercitrin, myricitrin, nicotiflorin, quercitrin, phlorizin, myricetin, trilobatin, eriodictyol, quercetin, luteolin, naringenin, phloretin, kaempferol, and procyanidin, respectively, by comparing the data with those of authentic standards.

Compounds 64 and 88 possessed the same quasi-molecular ions and characteristic fragment ions as compound 97; thus, they were characterized as quercetin 3-O-rutinoside isomers. Similarly, compounds 84, 114, and 139 were isoquercitrin isomers, and compounds 109, 140, 146, and 154 were assigned as isomers of nicotiflorin, quercetin, myricetin, and luteolin, respectively. Compounds 110 and 119 were tentatively presumed to be phlorizin isomers.

Compound 51, with the deprotonated ion [M-H]^−^ at *m/z* 625.1413, was eluted at 6.08 min, with the main characteristic fragment ion at *m/z* 463.0877, owing to the loss of a glucose residue (162 Da), which further gave rise to product ions at *m/z* 301.0351. It was tentatively characterized as a quercetin 3,4′-diglucoside [13]. Likewise, compounds 74, 92, 124, and 148 were deduced to be quercetin derivatives; compound 78 was quercetin 3-rutinoside 7-rhamnoside [14]; and compounds 100 and 108 were quercetin 3-O-(6″-galloyl)-*β*-D-glucopyranoside isomers. Compound 105 was characterized as quercitrin 3-O-glucuronide, and compounds 116 and 122 were quercitrin 3-O-arabinoside isomers [15–17].

Compounds 53, 69, and 89 exhibited quasi-molecular ions [M-H]^−^ at *m/z* 303.0510, and fragment ions at *m/z* 125.0232, 151.0026, and 177.0187 were tentatively characterized as taxifolin isomers, as previously reported [16].

Compound 57 was found at 6.23 min, yielded a parent ion [M-H]^−^ at *m/z* 609.1461 consisting of kaempferol (285 Da) and two glucose moieties (324 Da), and was identified as kaempferol 3,7-diglucoside [13].

Similarly, compound 141 was kaempferol-7-O-rhamnoside, and compounds 35, 45, 70, and 76 were suggested to be kaempferol derivatives [18].

Compounds 48, 71, 85, and 101 were detected at 5.95, 6.69, 7.17, and 7.70 min, respectively, and possessed the same quasi-molecular ions [M-H]^−^ at *m/z* 641.1359, MS/MS fragment ions at *m/z* 317.0301, and 316.0200 owing to the loss of two galactoside residues (324 Da), indicating the presence of a myricetin group. Therefore, they have been characterized as myricetin 3,3′-digalactoside isomers. Likewise, compounds 68, 82, 83, 93, and 138 were assigned as myricetin 3-rutinoside-7-rhamnoside, myricetin 3-O-glucuronide, myricetin 3-O-galactoside, myricetin derivative, and myricetin 3-O-(6″-galloyl)-*β*-D-rhamnoside, respectively, and compounds 73, 79, and 106 were myricetin 3-O-rutinoside isomers [19–21].

Compounds 50 and 65 were eluted at 6.07 and 6.53 min, respectively, and possessed the same quasi-molecular ion [M-H]^−^ at *m/z* 319.0459 and fragment ions at *m/z* 125.0232, 193.0134, and 151.0026. They were tentatively assigned as dihydromyricetin isomers by referring to the literature [22].

Compounds 52 and 60 yielded a quasi-molecular ion [M-H]^−^ at *m/z* 449.1089, which was tentatively identified as the maesopsin 4-O-glucoside isomer according to a previously published paper [21]. Likewise, compounds 77, 99, 149, and 158 were catechin di-C-hexoside, 3′, 5′-di-C-*β*-d-glucosylphloretin [23], chrysin derivatives, and chrysoeriol, respectively. Compounds 135 and 143 were 5,2′,6′-dihydroxy-7,8-dimethoxyflavone isomers [24], and compounds 132 and 133 were viscidulin III 6′-o-*β*-d-glucoside isomers [24].

Compounds 62 and 123 showed a deprotonated ion [M-H]^−^ at *m/z* 287.0561. The appearance of fragment ions at *m/z* 125.0233 and 151.0027 in the MS^2^ spectrum of those compounds indicated that they were (2S)-5,7,2′,6′-tetrahydroxyflavanone isomers [24].

Compounds 66, 91, and 103 yielded a quasi-molecular ion [M-H]^−^ at *m/z* 433.1140 and were eluted at 6.58, 7.44, and 7.73 min, respectively, which showed fragment ions at *m/z* 271.0611 by the neutral loss of glucose moieties (162 Da). Thus, they were considered to be naringenin 7-O-glucoside isomers [25]. Similarly, compounds 102 and 118 were naringenin-O-glucoside-rhamnosides.

Compounds 75 and 81 appeared at a retention time (*t*_*R*_) of 6.96 and 7.07 min, respectively, possessing the quasi-molecular ions [M-H]^−^ at *m/z* 433.1140 and their fragment ions at *m/z* 313.0717 ([M-H-120]^−^) and 343.0820 ([M-H-90]^−^), which were identified as naringenin 6-C-glucosidei somer [26]. Similarly, compound 47 was deduced as naringenin-6,8-di-C-glucoside [27].

Compounds 152 and 156 were found at 12.34 and 12.97 min, respectively, which show the common precursor ion [M-H]^−^ at *m/z* 315.0505, and the major fragment ion at *m/z* 300.0275 due to loss of a CH_3_ reside (15 Da), then they were tentatively characterized as isorhamnetinisomer [17]. Compounds 112, 126, 127, 137, and 142 appeared at retention times (*t*_*R*_) of 8.10, 8.72, 8.87, 9.58, and 10.00 min, respectively, which were tentatively identified as 3-methylquercetin-7-O-glucoside isomers. The parent ions at *m/z* 477.1038 were due to the loss of glucose moieties (162 Da) and generated the characteristic fragment ions at *m/z* 315.0505 [17].

#### 3.4.2. Identification of Phenylpropanoids in *Diospyros lotus* L

Compounds 30, 40, 42, 67, 87, 111, 115, and 136 were eluted at 3.33, 5.42, 5.46, 6.65, 7.29, 8.04, 8.28, and 9.45 min, respectively. They were characterized as neochlorogenic acid, chlorogenic acid, caffeic acid, isochlorogenic acid B, ferulic acid, 1,3-dicaffeoylquinic acid, isochlorogenic acid A, and isochlorogenic acid C, respectively, by comparison to commercial reference standards.

Compound 61 possessed the same quasi-molecular ions, and the characteristic fragment ion of compound 42 was characterized as a caffeic acid isomer. Similarly, compounds 95, 104, and 129 were ferulic acid isomers.

Compounds (56, *t*_*R*_ 6.22 min, and 63, *t*_*R*_ 6.52 min) had the same quasi-molecular ions [M-H]^−^ at *m/z* 355.1035 and the fragment ion at *m/z* 193.0500, corresponding to the neutral loss of the glucose group (162 Da) and further generation of the fragment ions of compound 87. Therefore, they were tentatively assigned as ferulic acid acyl-*β*-D-glucoside isomers [17].

Compounds 37, 43, 44, and 46 were eluted at 5.30, 5.49, 5.60, and 5.73 min, respectively, and showed a deprotonated molecular ion [M-H]^−^ at *m/z* 457.1351. They were tentatively inferred to be p-coumaric acid-O-glucoside-rhamnoside based on the base peak ion in the MS^2^ spectrum.

Compounds 80, 90, and 121 were eluted at 7.07, 7.41, and 8.42 min, respectively, yielding a deprotonated ion [M-H]^−^ at *m/z* 579.2083 and fragment ions at *m/z* 417.1554, 181.0497, and 402.1317, which were suggested as syringaresinol O-*β*-D-glucoside isomers in comparison with the literature [28].

Compounds 38, 59, and 96 were eluted at 5.36, 6.39, and 7.60 min, respectively, and yielded the same parent ion [M-H]^−^ at *m/z* 177.0193. They were deduced as esculetin isomers according to the MS and MS/MS spectra [25].

#### 3.4.3. Identification of Organic Acids in *Diospyros lotus* L

Compounds 2, 5, and 10 were found at 0.86, 0.94, and 1.34 min, respectively, and possessed the same parent ion [M-H]^−^ at *m/z* 191.0561. Compound 5 was identified as quinic acid by comparison with the reference substances. Thus, compounds 2 and 10 were identified as isomers of quinic acid.

Compounds 1 and 4 were observed at 0.83 and 0.94 min, respectively, and possessed the same quasi-molecular ions [M-H]^−^ at *m/z* 93.0347 and MS/MS fragment ions at *m/z* 71.0124, 101.0230, and 113.0231. They were tentatively assigned as glucuronic acid isomers by comparison to the literature. Similarly, compounds 3, 6, and 8 were citric acid isomers [20], compounds 11 and 22 were 3-methylglutaric acid isomers, compound 27 was syringic acid glucoside [29], compounds 17 and 18 were pantothenic acid isomers, compound 55 was dihydrophaseic acid, compound 120 was azelaic acid, and compounds 130 and 145 were abscisic acid isomers [16, 27].

Compound 14 exhibited a quasi-molecular ion [M-H]^−^ at *m/z* 169.0142 and generated the main characteristic fragments ion at *m/z* 125.0233 ([M-CO_2_-H]^−^), which was identified as gallic acid by the MS and MS/MS spectra [30]. Compounds 7, 12, 13, and 21, with the same deprotonated ions [M-H]^−^ at *m/z* 331.0671, were eluted at 1.07, 1.36, 1.41, and 1.91 min, respectively. The main fragment ions, at *m/z* 169.0132, were obtained by the loss of glucose moieties (162 Da) as well as characteristic fragment ions of gallic acid (m/z 125.0233), which were deduced as 6-O-galloylglucose isomers [14]. Similarly, compound 9 was identified as 6-O-galloylsucrose.

Compound 33 was eluted at 4.07 min, possessing a quasi-molecular ion [M-H]^−^ at *m/z* 183.0298, showing characteristic fragment ions at (m/z 140.0103, 124.0153), and was characterized as methyl gallate [16].

Compounds 15 and 39 were detected at 1.65 and 5.37 min, respectively. They showed the same deprotonated ion [M-H]^−^ at *m/z* 153.0193 and the fragment ions at *m/z* 108.0203, 109.0282, and 123.0439, suggesting that they were 2,3-dihydroxybenzoic acid isomers [27, 31]. Compound 20 yielded a deprotonated ion [M-H]^−^ at *m/z* 315.0722, which showed a fragment ion at *m/z* 153.0186 by losing the glucose moiety (162 Da) in the MS^2^ fragment ions; therefore, it was tentatively identified as a 2,3-dihydroxybenzoic acid 3-O-glucoside isomer [31].

Compounds 16, 19, 28, 29, 41, 58, 113, and 131 were detected at 1.67, 1.84, 3.09, 3.19, 5.44, 6.38, 8.10, and 9.18 min, respectively, and possessed the same quasi-molecular ions [M-H]^−^ at *m/z* 151.0401. The characteristic fragment ions at *m/z* 108.0204, 123.0439, and 136.0154 were identified as vanillin isomers according to the base peaks and retention times. Compounds 72 and 86 were found at 6.72 and 7.23 min, respectively, and yielded the parent ions [M-H]^−^ at *m/z* 167.0350. They were identified as vanillic acid isomers based on the MS and MS/MS spectra [16]. Compound 49 appeared at a *t*_*R*_ of 5.95 min, possessing quasi-molecular ions at *m/z* 329.0878 and the main fragment ion at *m/z* 167.0341 owing to the loss of a glucose residue (162 Da), which was characterized as vanillic acid glucoside [32]. Similarly, compounds 34 and 54 were confirmed as vanillic acid-O-rutinosides.

Compound 94 at *m/z* 137.0244 with the molecular formula C_7_H_6_O_3_ and appearing at a *t*_*R*_ of 7.58 min was suggested to be p-hydroxybenzoic acid based on the MS^2^ data [21]. Compounds 23, 24, 25, 26, 31, and 32 (*t*_*R*_ 2.65, 2.85, 2.89, 3.00, 3.53, and 3.75 min, respectively) had the same quasi-molecular ion [M-H]^−^ at *m/z* 299.0772 and the characteristic fragment ion at *m/z* 137.0233 based on the neutral loss of a glucose residue (162 Da). They were tentatively characterized as p-hydroxybenzoic acid-O-glucoside isomers [33].

### 3.5. Active Components and Related Targets

**Figure 2:**
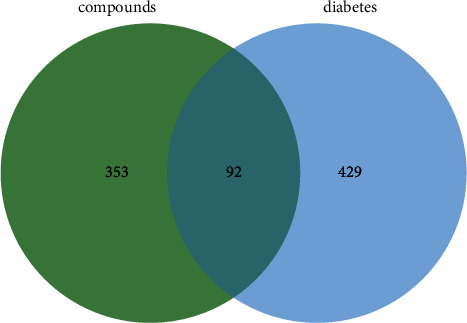
Overlapping genes of diabetes and compound targets.

The active compounds were selected by using the “drug-like soft” in FAFDrugs4 with the criteria of 100 ≤ MW ≤ 600, −3 ≤ logP ≤ 6, and HBA ≤ 12. Eventually, a total of 40 compounds were screened (Supplementary Table 1). Combined with TCMSP and STP database search and prediction, 445 component targets were obtained after removing duplicate targets. Furthermore, 521 diabetes-related targets were identified by screening the disease-target database. Finally, 92 overlapping genes of compound targets and diabetes-related targets were regarded as potential targets of *Diospyros lotus* L for the treatment of diabetes (Figure 2).

### 3.6. PPI Network of Overlapping Genes

The PPI network graph was obtained by importing 92 overlapping targets into STRING and removing one disconnected point. There were 91 nodes and 1488 edges; the average number of nodes was 32.8, and the average local clustering coefficient was 0.703. TSV data were downloaded and imported into Cytoscape 3.9.0 software to show the protein interaction network.

**Figure 3:**
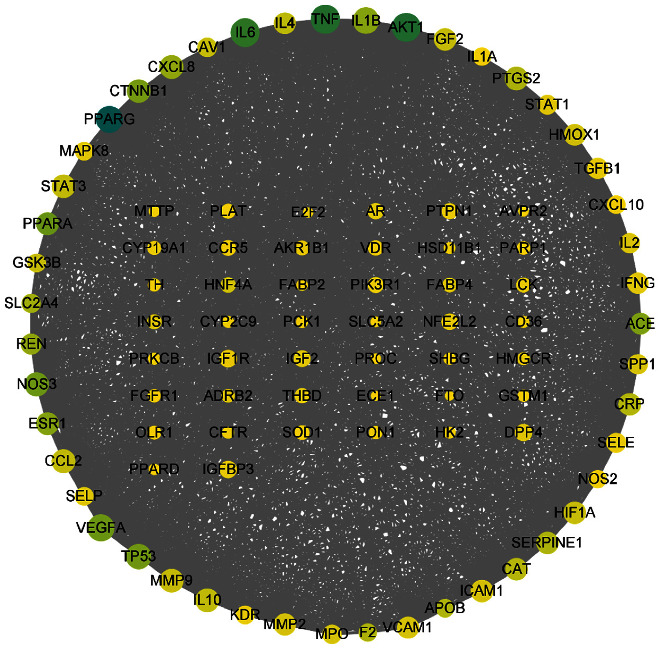
PPI network graph.

The results are shown in Figure 3, where the node size is positively correlated with the degree value and the lines represent interactions. As betweenness centrality increases, the color of the node changes from yellow to turquoise. Degree and betweenness centrality indicate the importance of the targets. The target whose degree value was greater than the average value was considered the key target.

### 3.7. Enrichment Analysis

The key targets were further analyzed by functional association clustering to integrate functional genomics annotations of the most important cluster of targets and pathways, which facilitates further understanding of the mechanism of the antidiabetic effect of *Diospyros lotus* L.

**Figure 4:**
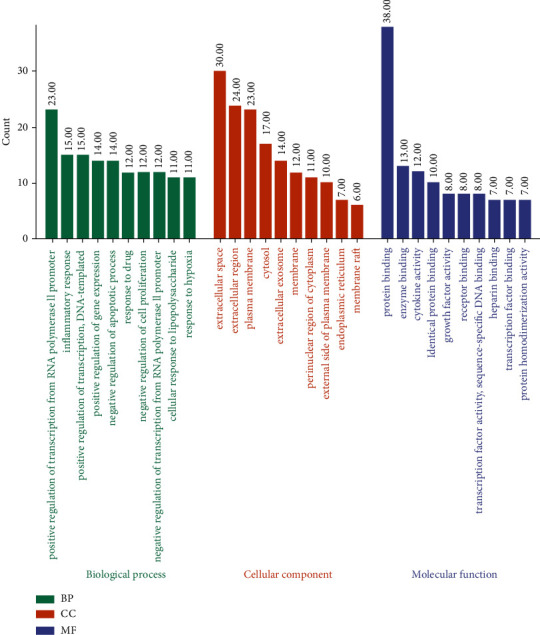
Top 10 in GO analysis.

As shown in Figure 4, the most representative GO-BP terms were “positive regulation of transcription from RNA polymerase II promoter” and “inflammatory response,” whereas the most representative GO-CC terms were “extracellular space,” “extracellular region,” and “plasma membrane.” The most representative GO-MF terms were “protein binding” and “enzyme binding.”

**Figure 5:**
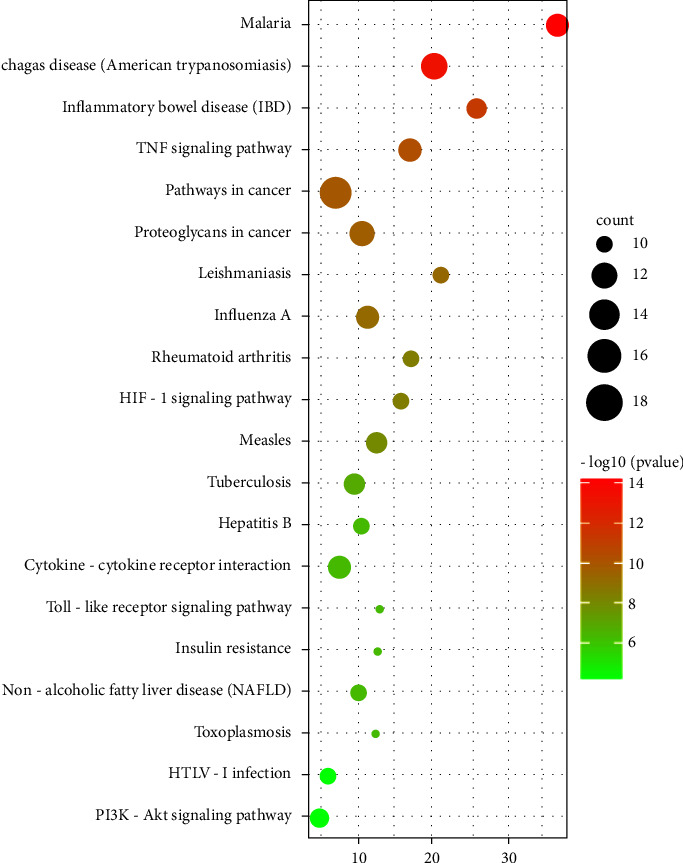
Significant pathway enrichment bubble diagram (top 20).

The two most representative KEGG pathways (Figure 5) were the “MAPK signaling pathway” and “AGE-RAGE signaling pathway.” After exclusion of broad pathways, 47 core common target genes were mainly related to the TNF, PI3K-Akt, HIF-1, NAFLD, toll-like receptor, and other multiple signaling pathways. This suggests that the effect of *Diospyros lotus* L on diabetes may involve multiple pathways as well as complex interactions among these pathways.

### 3.8. Active Component-KeyGene-Pathway Interaction Network Analysis

**Figure 6:**
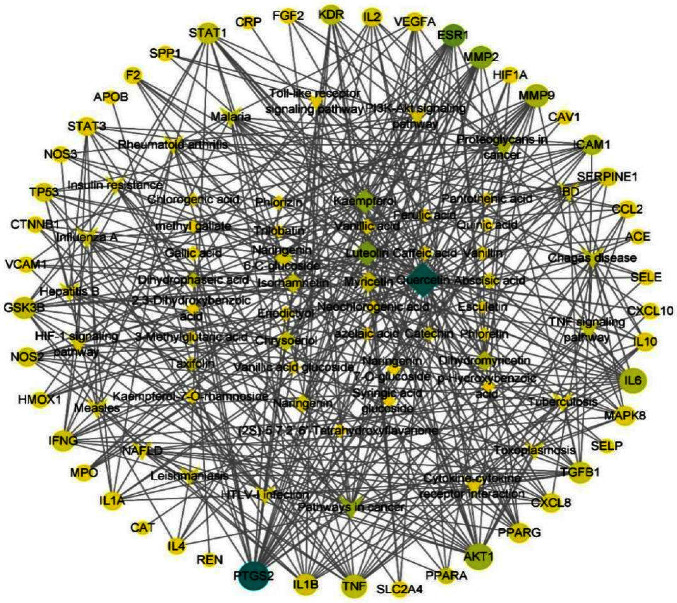
The active component-keygene-pathway interaction network. As the betweenness centrality increases, the color of the node changes from yellow to turquoise, and the larger the node, the greater the degree value.

As shown in Figure 6, the active component-keygene-pathway interaction network contained 104 nodes (47 key genes, 37 active components, and 20 KEGG pathways (top 20)) and 410 edges. In the network, the diamond, oval, and elliptical nodes correspond to different active compounds, pathways, and targets, respectively. The degrees of quercetin, luteolin, kaempferol, TNF signaling pathway, PI3K-Akt signaling pathway, HIF-1 signaling pathway, PTGS2, AKT1, IL6, and TNF were 34, 20, 18, 12, 11, 10, 25, 21, 20, and 17, respectively. The average degrees of the diamond and elliptical nodes were 5.93° and 8.72°, respectively. In addition, at least nine genes were potentially involved in each diabetes-related pathway, suggesting that one active component can potentially target multiple genes and have the action characteristics of multiple active compounds, targets, and pathways of *Diospyros lotus* L in the treatment of diabetes.

Quercetin has many antihyperglycemic effects, such as enhancing insulin sensitivity, promoting glycogen synthesis, inhibiting *α*-glucosidase activity, and improving insulin resistance [34]. Luteolin can play an antioxidant role by enhancing the activity of superoxide dismutase in microvascular lesions in diabetes [35]. Kaempferol is a flavonoid compound that plays an active role in the prevention and treatment of diabetes and has anti-inflammatory and antioxidant properties. It can reduce oxidative stress and inflammation through the MAPK pathway to alleviate myocardial ischemia-reperfusion injury in diabetic rats [36]. Myricetin can enhance the antioxidant defense system in mice, increase insulin secretion, substantially reduce blood glucose levels, and effectively protect the liver and kidney from oxidative damage in diabetic mice [37, 38]. IL-6 interferes with the insulin signaling pathway and promotes apoptosis of pancreatic *β*-cells, which promotes insulin resistance in multiple organs through a variety of inflammatory signaling pathways [39]. TNF is one of the cytokines constituting the acute inflammatory response, which can trigger the MAPK and NF-*κ*B pathways, leading to insulin resistance [40].

## 4. Conclusion

In this study, an integrated approach combining UHPLC-Q-Exactive Orbitrap MS and network pharmacology analysis was adopted to explore the potential active ingredients and ameliorative mechanisms of *Diospyros lotus* L against hyperglycemia. Eventually, 159 compounds were identified in *Diospyros lotus* L (140 of which were reported for the first time). According to the results of the active components and key gene-pathway interaction network, the antihyperglycemic effect of *Diospyros lotus* L is attributed to quercetin, luteolin, kaempferol, myricetin, and dihydromyricetin, which act on PTGS2, AKT1, IL6, TNF, and MMP9 and participate in the TNF, PI3K-Akt, and HIF-1 signaling pathways, as well as NAFLD. In conclusion, the integrated approach combining UHPLC-Q-Exactive Orbitrap MS and network pharmacology analysis provided insights into the potential active ingredients and ameliorative mechanism of *Diospyros lotus* L on hyperglycemia.

## Figures and Tables

**Table 1:** The range of the parameters of the “drug-like soft” principle.

Property	Range	Property	Range
MW	100–600	logP	−3–6
HBA	≤12	HBD	≤7
tPSA	≤180	Rotatable bonds	≤11
Rigid bonds	≤30	Rings	≤6
Max size system ring	≤18	Carbons	3–35
Hetero atoms	1–15	H/C ratio	0.1–1
Charges	≤4	Total charge	−4–4

**Table 2:** The chromatographic and mass data of detected components from *Diospyros lotus* L through UHPLC-Q-Exactive Orbitrap MS.

Peak	*t*_*R*_	Theoretical mass (*m/z*)	Experimental mass (*m/z*)	Error (ppm)	Formula	MS/MS fragment	Identification	Peak area
1	0.83	193.0347	193.0346	−4.23	C_6_H_10_O_7_	MS^2^[193]: 71.0124(100), 101.0230(5), 113.0231(2)	Glucuronic acid isomer	423409833
2	0.86	191.0561	191.0554	−3.72	C_7_H_12_O_6_	MS^2^[191]:85.0282(100),111.0076(76), 87.0074(34)	Quinic acid isomer	30408553
3	0.90	191.0197	191.0190	−3.90	C_6_H_8_O_7_	MS^2^[191]: 111.0075(100)	Citric acid isomer	2270126001
4	0.94	193.0347	193.0347	−3.76	C_6_H_10_O_7_	MS^2^[193]: 71.0125(100), 113.0232(82), 101.0232(40)	Glucuronic acid isomer	465384616
5	0.94^*∗*^	191.0561	191.0544	−9.06	C_7_H_12_O_6_	MS^2^[191]: 111.0076(100), 87.0074(53), 85.0282(29)	Quinic acid	65213188
6	1.05	191.0197	191.0189	−4.21	C_6_H_8_O_7_	MS^2^[191]: 111.0076(100)	Citric acid isomer	14530674970
7	1.07	331.0671	331.0671	−0.06	C_13_H_16_O_10_	MS^2^[331]: 169.0132(100), 125.0231(71)	6-O-galloylglucose isomer	3462772
8	1.13	191.0197	191.0190	−3.90	C_6_H_8_O_7_	MS^2^[191]: 111.0076(100)	Citric acid isomer	26089906774
9	1.30	493.1199	493.1200	0.22	C_19_H_26_O_15_	MS^2^[493]: 169.0132(100), 331.0669(93)	6-O-galloylsucrose	105557866
10	1.34	191.0561	191.0552	−5.03	C_7_H_12_O_6_	MS^2^[191]: 111.0076(100), 87.0075(49), 85.0282(27)	Quinic acid isomer	49104868
11	1.35	145.0506	145.0495	−7.81	C_6_H_10_O_4_	MS^2^[145]: 57.0332(100), 71.0488(24), 83.0489(23)	3-Methylglutaric acid isomer	55463923
12	1.36	331.0671	331.0672	0.30	C_13_H_16_O_10_	MS^2^[331]: 169.0132(100), 125.0231(65)	6-O-galloylglucose isomer	109327970
13	1.41	331.0671	331.0671	0.03	C_13_H_16_O_10_	MS^2^[331]: 169.0132(100), 125.0231(64)	6-O-galloylglucose isomer	105149835
14	1.48	169.0142	169.0134	−4.83	C_7_H_6_O_5_	MS2[169]: 125.0233(100)	Gallic acid	663649505
15	1.65	153.0193	153.0184	−6.35	C_7_H_6_O_4_	MS^2^[153]: 108.0203(100), 109.0282(74), 123.0439(6)	2,3-Dihydroxybenzoic acid isomer	88754053
16	1.67	151.0401	151.0391	−6.67	C_8_H_8_O_3_	MS^2^[151]: 108.0204(100), 124.0153(51), 123.0438(31), 136.0155(16)	Vanillin isomer	14956041
17	1.69	218.1030	218.1029	−2.18	C_9_H_17_NO_5_	MS^2^[218]: 88.0391(100), 146.0813(53)	Pantothenic acid isomer	692269068
18	1.76	218.1030	218.1029	−2.18	C_9_H_17_NO_5_	MS^2^[218]: 88.0391(100), 146.0813(53)	Pantothenic acid isomer	559182584
19	1.84	151.0401	151.0391	−6.54	C_8_H_8_O_3_	MS^2^[151]: 108.0204(100), 124.0154(45), 123.0439(34), 136.0154(11)	Vanillin isomer	15928418
20	1.84	315.0722	315.0724	0.65	C_13_H_16_O_9_	MS^2^[315]: 108.0203(100), 109.0281(43), 153.0186(18), 123.0439(9)	2,3-Dihydroxybenzoic acid 3-O-glucoside isomer	13894787
21	1.91	331.0671	331.0673	0.67	C_13_H_16_O_10_	MS^2^[331]: 125.0231(100), 169.0132(94)	6-O-galloylglucose isomer	6714540
22	2.31	145.0506	145.0497	−6.77	C_6_H_10_O_4_	MS^2^[145]: 83.0489(30), 101.0594(10), 57.0332(7), 71.0124(6)	3-Methylglutaric acid isomer	37732233
23	2.65	299.0772	299.0770	−0.70	C_13_H_16_O_8_	MS^2^[137]: 137.0233(100), 93.0332(78)	P-hydroxybenzoic acid-O-glucoside isomer	5442116
24	2.85	299.0772	299.0773	0.30	C_13_H_16_O_8_	MS^2^[137]: 137.0233(100), 93.0332(78)	P-hydroxybenzoic acid-O-glucoside isomer	5284965
25	2.89	299.0772	299.0773	0.10	C_13_H_16_O_8_	MS^2^[137]: 137.0233(100), 93.0332(78)	P-hydroxybenzoic acid-O-glucoside isomer	5801580
26	3.00	299.0772	299.0775	0.80	C_13_H_16_O_8_	MS^2^[137]: 137.0233(100), 93.0332(66)	P-hydroxybenzoic acid-O-glucoside isomer	8954168
27	3.00	359.0984	359.0989	1.59	C_15_H_20_O_10_	MS^2^[359]: 138.0311(100), 182.0211(86), 197.0447(44), 153.0546(38)	Syringic acid glucoside	16332217
28	3.09	151.0401	151.0391	−6.14	C_8_H_8_O_3_	MS^2^[151]: 108.0232(100), 124.0154(37), 123.0441(14), 136.0153(11)	Vanillin isomer	27511009
29	3.19	151.0401	151.0391	−6.33	C_8_H_8_O_3_	MS^2^[151]:108.0202(100), 124.0153(31), 123.0439(23), 136.0153(23)	Vanillin isomer	23855779
30	3.33^*∗*^	353.0878	353.0880	0.67	C_16_H_18_O_9_	MS^2^[353]: 191.0551(100), 135.0443(10)	Neochlorogenic acid	371210
31	3.53	299.0772	299.0770	−0.70	C_13_H_16_O_8_	MS^2^[137]: 137.0232(100), 93.0332(90)	P-hydroxybenzoic acid-O-glucoside isomer	6350760
32	3.75	299.0772	299.0771	−0.60	C_13_H_16_O_8_	MS^2^[137]: 93.0332(100), 137.0233(85)	P-hydroxybenzoic acid-O-glucoside isomer	4495564
33	4.07	183.0299	183.0295	−3.86	C_8_H_8_O_5_	MS^2^[183]: 140.0103(100), 124.0153(78), 111.0074(65), 139.0025(53)	Methyl gallate	54964007
34	4.93	475.1457	475.1455	−0.49	C_20_H_28_O_13_	MS^2^[475]: 167.0340(100), 123.0439(56)	Vanillic acid-O-rutinoside	5156267
35	4.96	465.1038	465.1035	−0.86	C_21_H_22_O_12_	MS^2^[465]: 285.0403(100), 125.0232(67), 151.0028(11)	Kaempferol derivative	2220696
36	4.99^*∗*^	289.0718	289.0726	3.01	C_15_H_14_O_6_	MS^2^[289]:109.0282(100), 123.0433(70), 97.0288(32), 125.0231(30)	Catechin	1491579
37	5.30	457.1351	457.1354	0.64	C_20_H_26_O_12_	MS^2^[457]: 119.0491(100), 163.0391(58)	P-coumaric acid-O-glucoside-rhamnoside	12791866
38	5.36	177.0193	177.0186	−4.19	C_9_H_6_O_4_	MS^2^[177]: 133.0284(100), 105.0334(64), 89.0383(31), 149.0234(16)	Esculetin isomer	68123540
39	5.37	153.0193	153.0184	−6.16	C_7_H_6_O_4_	MS^2^[153]: 109.0282(100), 108.0202(19)	2,3-Dihydroxybenzoic acid isomer	263921011
40	5.42^*∗*^	353.0878	353.0880	0.58	C_16_H_18_O_9_	MS^2^[353]: 191.0554(100), 135.0441(12)	Chlorogenic acid	36538266
41	5.44	151.0401	151.0391	−6.27	C_8_H_8_O_3_	MS^2^[151]: 108.0204(100), 123.0439(35), 124.0154(32), 136.0152(23)	Vanillin isomer	16864944
42	5.46^*∗*^	179.0350	179.0345	−2.86	C_9_H_8_O_4_	MS^2^[179]: 135.04401(100), 179.03407(87)	Caffeic acid	12320409
43	5.49	457.1351	457.1356	1.03	C_20_H_26_O_12_	MS^2^[457]: 119.0491(100), 163.0392(54)	P-coumaric acid-O-glucoside-rhamnoside	25886930
44	5.60	457.1351	457.1354	0.64	C_20_H_26_O_12_	MS^2^[457]: 119.0491(100), 163.0392(50)	P-coumaric acid-O-glucoside-rhamnoside	6494575
45	5.64	465.1038	465.1036	−0.58	C_21_H_22_O_12_	MS^2^[465]: 285.0403(100), 125.0233(54), 178.9976(17), 151.0029(9)	Kaempferol derivative	2286572
46	5.73	457.1351	457.1353	0.35	C_20_H_26_O_12_	MS^2^[457]: 119.0490(100), 163.0391(48)	P-coumaric acid-O-glucoside-rhamnoside	14719551
47	5.86	595.1663	595.1680	1.37	C_27_H_32_O_15_	MS^2^[595]: 355.0822(100), 385.0927(91), 415.1031(25), 475.1259(16)	Naringenin-6,8-di-C-glucoside	3936541
48	5.95	641.1359	641.1370	1.60	C_27_H_30_O_18_	MS^2^[641]: 479.0816(100), 178.9978(21), 151.0026(13)	Myricetin 3,3'-digalactoside isomer	907936
49	5.95	329.0878	329.0880	0.53	C_14_H_18_O_9_	MS^2^[329]: 167.0341(100), 123.0440(45)	Vanillic acid glucoside	184241354
50	6.07	319.0459	319.0461	0.34	C_15_H_12_O_8_	MS^2^[319]: 193.0136(100), 125.0233(77), 151.0028(17), 165.0184(15)	Dihydromyricetin isomer	42196138
51	6.08	625.1410	625.1413	0.44	C_27_H_30_O_17_	MS^2^[625]: 463.0877(100), 301.0351(60), 151.0025(15)	Quercetin 3,4'-Diglucoside	3023028
52	6.09	449.1089	449.1091	0.37	C_21_H_22_O_11_	MS^2^[449]: 259.0611(100), 269.0457(86), 151.0027(47), 287.0287(17)	Maesopsin 4-O-glucoside isomer	36415310
53	6.16	303.0510	303.0510	−0.12	C_15_H_12_O_7_	MS^2^[303]: 125.0232(100), 151.0026(10), 177.0187(5)	Taxifolin isomer	4492484
54	6.17	475.1457	475.1459	0.39	C_20_H_28_O_13_	MS^2^[475]: 167.0339(100), 123.0439(4)	Vanillic acid-O-rutinoside	21026003
55	6.21	281.1396	281.1396	0.62	C_15_H_22_O_5_	MS^2^[281]: 123.0803(100), 171.1169(83), 189.1278(22)	Dihydrophaseic acid	13845836
56	6.22	355.1035	355.1037	0.60	C_16_H_20_O_9_	MS^2^[355]: 134.0363(100), 193.0500(93), 149.0598(26), 178.0265(13)	Ferulic acid acyl-*β*-D-glucoside isomer	155512403
57	6.23	609.1461	609.1467	1.02	C_27_H_30_O_16_	MS^2^[609]: 447.0926(100), 285.0401(34)	Kaempferol 3,7-diglucoside	1288267
58	6.38	151.0401	151.0391	−6.14	C_8_H_8_O_3_	MS^2^[151]: 108.0203(100), 136.0154(23), 123.0437(6)	Vanillin isomer	34876373
59	6.39	177.0193	177.0186	−3.97	C_9_H_6_O_4_	MS^2^[177]: 129.0183(100), 133.0284(21), 89.0385(8)	Esculetin isomer	5548229
60	6.44	449.1089	449.1091	0.30	C_21_H_22_O_11_	MS^2^[449]: 259.0610(100), 269.0455(75), 287.0561(34), 151.0026(8)	Maesopsin 4-O-glucoside isomer	12513053
61	6.48	179.0350	179.0343	−3.64	C_9_H_8_O_4_	MS^2^[179]: 135.04428(100)	Caffeic acid isomer	10375972
62	6.50	287.0561	287.0561	−0.18	C_15_H_12_O_6_	MS^2^[287]:125.0232(100), 151.0026(13),161.0230(3)	(2S)-5,7,2',6′-tetrahydroxyflavanone isomer	7395938
63	6.52	355.1035	355.1036	0.52	C_16_H_20_O_9_	MS^2^[355]: 134.0363(100), 193.0500(89), 149.0598(30), 178.0266(18)	Ferulic acid acyl-*β*-D-glucoside isomer	194391098
64	6.52	609.1461	609.1473	1.92	C_27_H_30_O_16_	MS^2^[609]: 301.0354(100), 447.0933(43), 300.0265(5)	Quercetin 3-O-rutinoside isomer	7578929
65	6.53	319.0459	319.0459	−0.13	C_15_H_12_O_8_	MS^2^[319]: 125.0232(100), 193.0134(75), 151.0026(20), 165.0184(20)	Dihydromyricetin isomer	3940259
66	6.58	433.1140	433.1144	0.76	C_21_H_22_O_10_	MS^2^[433]: 271.0611(100), 165.0183(30), 113.0231(12)	Naringenin 7-O-glucoside isomer	1556330
67	6.65^*∗*^	515.1195	515.1184	−2.12	C_25_H_24_O_12_	MS^2^[515]: 173.0447(100), 179.0342(88), 191.0554(38), 135.0441(15)	Isochlorogenic acid B	672858
68	6.67	771.1989	771.1996	0.84	C_33_H_40_O_21_	MS^2^[771]: 316.0225(100), 271.0247(47), 151.0026(8), 317.0232(4)	Myricetin 3-rutinoside 7-rhamnoside	37547695
69	6.67	303.0510	303.0511	0.31	C_15_H_12_O_7_	MS^2^[303]: 125.0233(100), 285.0408(47), 177.0183(11), 151.0027(8)	Taxifolin isomer	1258429
70	6.68	465.1038	465.1030	−1.76	C_21_H_22_O_12_	MS^2^[465]: 285.0404(100), 125.0232(57), 178.9976(18), 151.0027(6)	Kaempferol derivative	3746637
71	6.69	641.1359	641.1365	0.94	C_27_H_30_O_18_	MS^2^[641]: 479.0829(100), 317.0301(17), 316.0219(16), 151.0026(2)	Myricetin 3,3′-digalactoside isomer	8628387
72	6.72	167.0350	167.0341	−5.34	C_8_H_8_O_4_	MS^2^[167]: 123.0440(100), 108.0204(14)	Vanillic acid isomer	22418279
73	6.81	625.1410	625.1408	−0.44	C_27_H_30_O_17_	MS^2^[625]: 316.0224(100), 317.0266(19), 463.0877(8)	Myricetin 3-O-rutinoside isomer	1981287
74	6.88	423.0416	423.0393	−5.51	C_14_ H_16_ O_15_	MS^2^[423]: 151.0026(100), 178.9978(76), 301.0353(41)	Quercitrin derivative	41312151
75	6.96	433.1140	433.1137	−0.72	C_21_H_22_O_10_	MS^2^[433]: 313.0718(100), 343.0821(24), 271.0613(20), 151.0026(12)	Naringenin 6-C-glucoside isomer	2813357
76	6.96	465.1038	465.1032	−1.44	C_21_H_22_O_12_	MS^2^[465]: 161.0446(100), 125.0232(57), 285.0406(29)	Kaempferol derivative	1790280
77	7.01	613.1779	613.1779	0.74	C_27_H_34_O_16_	MS^2^[613]: 373.0929(100), 403.1037(56), 239.0556(27), 433.1139(17), 493.1359(17)	Catechin di-C-hexoside	18384963
78	7.03	755.2040	755.2045	0.60	C_33_H_40_O_20_	MS^2^[755]: 300.0274(100), 301.0337(16), 178.9976(9), 271.0247(4)	Quercetin 3-rutinoside 7-rhamnoside	7686510
79	7.03	625.1410	625.1413	0.44	C_27_H_30_O_17_	MS^2^[625]: 316.0244(100), 271.0250(48), 317.0293(12), 151.0027(9)	Myricetin 3-O-rutinoside isomer	117356236
80	7.07	579.2083	579.2109	4.43	C_28_H_36_O_13_	MS^2^[579]: 417.1554(100), 181.0496(84), 402.1320(20)	Syringaresinol-O-*β*-D-glucoside isomer	1274845
81	7.07	433.1140	433.1140	−0.02	C_21_H_22_O_10_	MS^2^[433]: 313.0717(100), 271.0611(82), 343.0820(22), 151.0025(14)	Naringenin 6-C-glucoside isomer	5777367
82	7.08	493.0624	493.0625	0.25	C_21_H_18_O_14_	MS^2^[493]: 317.0302(100), 151.0027(45), 109.0284(11), 271.0252(8)	Myricetin 3-O-glucuronide	61411051
83	7.13	479.0831	479.0831	−0.09	C_21_H_20_O_13_	MS^2^[479]:316.0255(100), 271.0249(41), 317.0301(13), 151.0026(7)	Myricetin 3-O-galactoside	179167814
84	7.16	463.0882	463.0885	0.65	C_21_H_20_O_12_	MS^2^[463]: 301.0350(100), 300.0274(61)	Isoquercitrin isomer	10171047
85	7.17	641.1359	641.1353	−1.07	C_27_H_30_O_18_	MS^2^[641]: 479.0831(100), 317.0301(58), 316.0222(17),	Myricetin 3,3′-digalactoside isomer	2349023
86	7.23	167.0350	167.0341	−5.28	C_8_H_8_O_4_	MS^2^[167]: 123.0440(100), 167.0341(85), 108.0206(5), 152.0109(4)	Vanillic acid isomer	272148953
87	7.29^*∗*^	193.0506	193.050	−3.48	C_10_H_10_O_4_	MS^2^[193]: 134.0362(100), 178.0266(48), 149.0598(21)	Ferulic acid	13558093
88	7.40	609.1461	609.1458	−0.59	C_27_H_30_O_16_	MS^2^[609]: 300.0273(100), 301.0328(16), 151.0025(7)	Quercetin 3-O-rutinoside isomer	368931
89	7.40	303.0510	303.0511	0.11	C_15_H_12_O_7_	MS^2^[303]: 125.0232(100), 151.0026(12), 177.0184(8)	Taxifolin isomer	18082158
90	7.41	579.2083	579.2114	5.38	C_28_H_36_O_13_	MS^2^[579]: 417.1553(100), 181.0497(79), 402.1321(13)	Syringaresinol-O-*β*-D-glucoside isomer	1680286
91	7.44	433.1140	433.1141	0.21	C_21_H_22_O_10_	MS^2^[433]: 271.0611(100), 151.0026(64), 119.0490(11)	Naringenin 7-O-glucoside isomer	3568902
92	7.48	435.1297	435.1300	0.85	C_21_H_24_O_10_	MS^2^[435]: 301.0307(100), 300.0271(5), 151.0027(3)	Quercitrin derivative	3058494
93	7.50	423.0416	423.0394	−5.30	C_14_ H_16_ O_15_	MS^2^[423]: 317.0126(100), 125.0233(40), 151.0026(15), 285.0401(10)	Myricetin derivative	461694539
94	7.58	137.0244	137.0234	−7.50	C_7_H_6_O_3_	MS^2^[137]: 93.0333(100)	P-hydroxybenzoic acid	1954942346
95	7.60	193.0506	193.0499	−3.64	C_10_H_10_O_4_	MS^2^[193]: 134.0363(100), 149.0600(62), 178.0264(60)	Ferulic acid isomer	943379
96	7.60	177.0193	177.0186	−4.30	C_9_H_6_O_4_	MS^2^[177]: 129.0182(100), 89.0231(65), 133.0283(16)	Esculetin isomer	8590955
97	7.60^*∗*^	609.1461	609.1466	0.73	C_27_H_30_O_16_	MS^2^[609]: 300.0275(100), 301.0351(94), 151.0026(3)	Quercetin 3-O-rutinoside	60494749
98	7.60^*∗*^	463.0882	463.0886	0.84	C_21_H_20_O_12_	MS^2^[463]: 316.0224(100), 271.0248(46), 287.0198(26), 151.0028(12), 317.0302(3)	Myricitrin	47320337
99	7.62	597.1825	597.1829	0.71	C_27_H_34_O_15_	MS^2^[597]: 357.0983(100), 387.1088(77), 167.0340(44), 209.0451(40), 417.1192(18)	3′, 5′-Di-C-*β*-D-glucosylphloretin	279295311
100	7.64	615.0992	615.0997	0.83	C_28_H_24_O_16_	MS^2^[615]: 463.0883(100), 301.0352(27), 300.0278(1)	Quercetin 3-O-(6″-galloyl)-*β*-D-glucopyranoside isomer	16017622
101	7.70	641.1359	641.1364	0.74	C_27_H_30_O_18_	MS^2^[641]: 479.0830(100), 317.0301(23), 316.0200(3)	Myricetin 3,3′-digalactoside isomer	308863
102	7.72	579.1719	579.1717	−0.41	C_27_H_32_O_14_	MS^2^[579]: 253.0504(100), 271.0613(48), 417.1552(17)	Naringenin-O-glucoside-rhamnoside	1975268
103	7.73	433.1140	433.1139	−0.23	C_21_H_22_O_10_	MS^2^[433]: 227.0709(100), 271.0611(54)	Naringenin 7-O-glucoside isomer	3090379
104	7.76	193.0506	193.0499	−3.90	C_10_H_10_O_4_	MS^2^[193]: 134.0363(100), 178.0265(61), 149.0599(49)	Ferulic acid isomer	10013718
105	7.76	477.0675	477.0678	0.66	C_21_H_18_O_13_	MS^2^[477]: 301.0352(100), 151.0026(12), 255.0296(1), 300.0270(1)	Quercitrin 3-O-glucuronide	71847819
106	7.78	625.1410	625.1418	1.21	C_27_H_30_O_17_	MS^2^[625]: 463.0882(100), 301.0350(29), 316.0223(11), 317.0289(6)	Myricetin 3-O-rutinoside isomer	3973219
107	7.82^*∗*^	463.0882	463.0888	1.25	C_21_H_20_O_12_	MS^2^[463]: 300.0276(100), 271.0250(68), 301.0353(47), 255.0299(31), 151.0027(16)	Isoquercitrin	193168329
108	7.92	615.0992	615.0994	0.35	C_28_H_24_O_16_	MS^2^[615]: 463.0883(100), 301.0353(26)	Quercetin 3-O-(6″-galloyl)-*β*-D-glucopyranoside isomer	2311932
109	8.00	593.1512	593.1518	1.01	C_27_H_30_O_15_	MS^2^[593]: 284.0325(100), 285.0400(62), 151.0025(6)	Nicotiflorin isomer	6641091
110	8.00	435.1297	435.1299	0.55	C_21_H_24_O_10_	MS^2^[435]: 167.0340(100), 273.0768(56), 125.0233(18), 123.0439(9)	Phlorizin isomer	7020357
111	8.04^*∗*^	515.1195	515.1201	1.21	C_25_H_24_O_12_	MS^2^[515]: 191.0553(100), 179.0341(92), 135.0440(14)	1,3-Dicaffeoylquinic acid	1538798
112	8.10	477.1038	477.1038	−0.12	C_22_H_22_O_12_	MS^2^[477]: 315.0510(100), 301.0352(95), 314.0434(15), 299.0197(14), 300.0272(10)	3-Methylquercetin 7-O-glucoside isomer	6541397
113	8.10	151.0401	151.0392	−5.54	C_8_H_8_O_3_	MS^2^[151]: 108.0204(100), 124.0125(24), 123.0440(16), 136.0153(13)	Vanillin isomer	11922688
114	8.21	463.0882	463.0884	0.46	C_21_H_20_O_12_	MS^2^[463]: 301.0352(100), 300.0277(2)	Isoquercitrin isomer	7397726
115	8.28^*∗*^	515.1195	515.1184	−2.12	C_25_H_24_O_12_	MS^2^[515]:191.0552(100), 179.0340(67), 353.0878(17), 135.0439(12)	Isochlorogenic acid A	3535566
116	8.31	433.1140	433.1142	0.42	C_21_H_22_O_10_	MS^2^[433]: 300.0273(100), 271.0610(77), 151.0026(32), 301.0332(26)	Quercitrin 3-O-arabinoside isomer	12897978
117	8.33^*∗*^	593.1512	593.1516	0.70	C_27_H_30_O_15_	MS^2^[593]: 285.0405(100), 284.0327(85), 255.0299(67), 227.0346(46)	Nicotiflorin	53519420
118	8.35	579.1719	579.1707	−2.09	C_27_H_32_O_14_	MS^2^[579]: 417.1554(100), 271.0607(28), 178.9978(12), 151.0025(14)	Naringenin-O-glucoside-rhamnoside	2699074
119	8.40	435.1297	435.1299	0.41	C_21_H_24_O_10_	MS^2^[435]: 167.0339(100), 273.0765(40), 125.0231(17)	Phlorizin isomer	6540475
120	8.40	187.0974	187.0967	−4.98	C_9_H_16_O_4_	MS^2^[187]: 125.0958(100), 97.0644(13), 169.0857(2)	Azelaic acid	1506853530
121	8.42	579.2083	579.2096	2.20	C_28_H_36_O_13_	MS^2^[579]: 417.1554(100), 181.0497(93), 402.1317(13)	Syringaresinol O-*β*-D-glucoside isomer	11293465
122	8.53	433.1140	433.1140	−0.09	C_21_H_22_O_10_	MS^2^[433]: 271.0612(100), 300.0274(34), 151.0027(30), 301.0331(11)	Quercitrin 3-O-arabinoside isomer	4688398
123	8.56	287.0561	287.0562	0.45	C_15_H_12_O_6_	MS^2^[287]: 125.0233(100), 151.0027(14)	(2S)-5,7,2′,6′-tetrahydroxyflavanone isomer	113424998
124	8.59	461.0725	461.0737	2.43	C_21_H_18_O_12_	MS^2^[461]:301.0353(100),178.9977(36), 151.0025(29), 300.0268(6)	Quercitrin derivative	5302276
125	8.67^*∗*^	447.0933	447.0935	0.50	C_21_H_20_O_11_	MS^2^[447]: 300.0276(100), 301.0353(71), 271.0249(64), 255.0298(44), 151.0027(25)	Quercitrin	314040631
126	8.72	477.1038	477.1036	−0.63	C_22_H_22_O_12_	MS^2^[477]: 314.0433(100), 315.0487(25), 301.0354(15), 300.0277(5), 299.0193(4)	3-Methylquercetin 7-O-glucoside isomer	2529387
127	8.87	477.1038	477.1039	0.19	C_22_H_22_O_12_	MS^2^[477]: 314.0432(100), 315.0489(23), 301.0352(6), 299.0194(5), 300.0273(3)	3-Methylquercetin 7-O-glucoside isomer	10332087
128	8.93^*∗*^	435.1297	435.1301	0.92	C_21_H_24_O_10_	MS^2^[435]:167.0341(100), 273.0760(39)	Phlorizin	715650
129	9.03	193.0506	193.0499	−3.79	C_10_H_10_O_4_	MS^2^[193]: 149.0598(100), 134.0361(25), 178.0264(16)	Ferulic acid isomer	6477816
130	9.13	263.1283	263.1289	0.22	C_15_H_20_O_4_	MS^2^[263]: 191.0343(100), 203.1072(5)	Abscisic acid isomer	3103377
131	9.18	151.0401	151.0392	−5.94	C_8_H_8_O_3_	MS^2^[151]: 108.0204(1000, 123.0439(17), 136.0156(14)	Vanillin isomer	11991089
132	9.20	507.1144	507.1150	1.08	C_23_H_24_O_13_	MS^2^[507]: 345.0611(100), 330.0367(25)	Viscidulin III 6′-O-*β*-D-glucoside isomer	2178417
133	9.29	507.1144	507.1144	0.05	C_23_H_24_O_13_	MS^2^[507]: 345.0610(100), 330.0367(31)	Viscidulin III 6′-O-*β*-D-glucoside isomer	573815
134	9.36^*∗*^	317.0303	317.0301	−0.63	C_15_H_10_O_8_	MS^2^[317]: 151.0026(100), 137.0233(73), 107.0125(33), 178.9977(31)	Myricetin	2396594
135	9.44	491.1195	491.1198	0.63	C_23_H_24_O_12_	MS^2^[491]: 313.0356(100), 271.0244(54), 299.0199(44), 329.0678(9)	5,2′6′-dihydroxy-7,8-dimethoxyflavone isomer	15215791
136	9.45^*∗*^	515.1195	515.1200	0.97	C_25_H_24_O_12_	MS^2^[515]: 179.0340(100), 191.0552(79), 353.0879(22), 135.0439(10)	Isochlorogenic acid C	3154984
137	9.58	477.1038	477.1040	0.32	C_22_H_22_O_12_	MS^2^[477]: 315.0514(100), 300.0273(26), 301.0351(21)	3-Methylquercetin 7-O-glucoside isomer	1113920
138	9.63	615.0992	615.0998	1.03	C_28_H_24_O_16_	MS^2^[615]: 317.0302(100), 463.0877(5), 316.0222(3), 178.9975(1)	Myricetin 3-O-(6″-galloyl)-*β*-D-rhamnoside	3665642
139	9.67	463.0882	463.0885	0.59	C_21_H_20_O_12_	MS^2^[463]: 301.0354(100), 300.0278(2), 255.0298(1)	Isoquercitrin isomer	31498699
140	9.82	301.0354	301.0355	0.28	C_15_H_10_O_7_	MS^2^[301]: 149.0234(100), 151.028(24), 107.0127(11), 121.0283(4)	Quercetin isomer	60769559
141	9.99	431.0983	431.0983	−0.23	C_21_H_20_O_10_	MS^2^[431]: 285.0405(100), 255.0298(84), 284.0328(74), 227.0346(64)	Kaempferol-7-O-rhamnoside	104050627
142	10.00	477.1038	477.1030	−1.78	C_22_H_22_O_12_	MS^2^[477]: 315.0510(100), 300.0275(36), 301.0347(21), 314.0753(11)	3-Methylquercetin 7-O-glucoside isomer	1327295
143	10.11	491.1195	491.1197	0.39	C_23_H_24_O_12_	MS^2^[491]: 328.0587(100), 329.0660(86), 313.0354(37), 299.0196(2)	5,2′6′-dihydroxy-7,8-dimethoxyflavone isomer	6215669
144	10.13^*∗*^	435.1297	435.1299	0.41	C_21_H_24_O_10_	MS^2^[435]: 273.0768(100), 167.0340(95), 125.0233(7), 123.0438(5)	Trilobatin	1544931
145	10.14	263.1283	263.1290	0.45	C_15_H_20_O_4_	MS^2^[263]: 203.1071(100), 191.0342(94), 151.0754(65), 152.0835(32)	Abscisic acid isomer	43256699
146	10.20	317.0303	317.0303	−0.65	C_15_H_10_O_8_	MS^2^[317]: 151.0026(100), 178.9976(33), 137.0233(14), 107.0126(7)	Myricetin isomer	6262287
147	10.33^*∗*^	287.0561	287.0561	−0.07	C_15_H_12_O_6_	MS^2^[287]: 135.0440(100), 151.0026(17)	Eriodictyol	6677796
148	11.00	505.0987	505.0993	1.00	C_23_H_22_O_13_	MS^2^[431]:301.0355(100), 151.0027(5), 300.0273(2)	Quercetin derivative	17504734
149	11.87	271.0612	271.0613	0.42	C_15_H_12_O_5_	MS^2^[271]: 143.0491(100), 253.0505(85), 209.0603(78)	Chrysin derivative	12485860
150	11.90^*∗*^	301.0354	301.0356	0.578	C_15_H_10_O_7_	MS^2^[301]: 149.0233(100), 151.027(30), 107.0127(11), 121.0286(5)	Quercetin	18626868
151	12.11^*∗*^	285.0405	285.0409	1.40	C_15_H_10_O_6_	MS^2^[285]: 133.0284(100), 151.0027(40), 175.0392(25), 199.0392(14)	Luteolin	1581682
152	12.34	315.0505	315.0511	0.30	C_16_H_12_O_7_	MS^2^[285]: 300.0275(100), 301.0309(16), 151.0028(1)	Isorhamnetinisomer	16370381
153	12.58^*∗*^	271.0612	271.0613	0.53	C_15_H_12_O_5_	MS^2^[271]: 119.0491(100), 151.0027(70), 177.0185(11),	Naringenin	129718757
154	12.59	285.0405	285.0410	1.86	C_15_H_10_O_6_	MS^2^[285]: 133.0283(100), 199.0396(48), 151.0027(19)	Luteolin isomer	3384989
155	12.82^*∗*^	273.0768	273.0773	1.55	C_15_H_14_O_5_	MS^2^[273]: 167.0342(100), 123.0456(39)	Phloretin	2148980
156	12.97	315.0505	315.0528	5.73	C_16_H_12_O_7_	MS^2^[285]: 300.0275(100), 301.0309(15)	Isorhamnetinisomer	3605778
157	13.19^*∗*^	285.0405	285.0407	0.98	C_15_H_10_O_6_	MS^2^[285]: 285.0405(100), 178.9917(43), 151.0027(16), 185.0602(16), 229.0503(13)	Kaempferol	9479859
158	13.51	299.0561	299.0564	0.87	C_16_H_12_O_6_	MS^2^[299]: 285.0325(100), 255.0298(43), 239.0347(40), 227.0344(17)	Chrysoeriol	3009700
159	14.46^*∗*^	593.1301	593.1305	0.72	C_30_H_26_O_13_	MS^2^[593]: 209.0449(100), 121.0283(70)	Procyanidin	3914568

^∗^identified by comparison with standard.

## Data Availability

The data used to support the finding of this study are available from the corresponding author upon request.

## Supplementary Materials

Supplementary Materials Supplementary Table 1: Results of screening compounds by “drug-likesoft” in FAFDrugs4.
