# Supplementary material for: Rapid Characterization and Action Mechanism of the Antidiabetic Effect of Diospyros lotus L Using UHPLC-Q-Exactive Orbitrap MS and Network Pharmacology
Source: J Anal Methods Chem. 2022 Dec 31;2022:8000126. doi: 10.1155/2022/8000126 (PMC9825215; doi:10.1155/2022/8000126)
Supplement: Supplementary Materials — Supplementary Table 1: Results of screening compounds by “drug-likesoft” in FAFDrugs4. [file 8000126.f1.docx]

Supplementary Table 1: results of screening compounds by “druglikesoft”in FAFDrugs 4

|  | compound | MW | logP | logD | logSw | tPSA | RotatableB | RigidB | Flexibility | HBD | HBA | HBD_HBA | Rings | MaxSize Ring | TotalCharge | HeavyAtoms | Result |
| --- | --- | --- | --- | --- | --- | --- | --- | --- | --- | --- | --- | --- | --- | --- | --- | --- | --- |
| 1 | Quinic acid | 192.17 | -2.37 | -6.08 | 0.53 | 121.05 | 1 | 7 | 0.13 | 5 | 6 | 11 | 1 | 6 | -1 | 13 | Accepted |
| 2 | 3-Methylglutaric acid isomer | 146.14 | 0.12 | -5.55 | -0.56 | 80.26 | 4 | 2 | 0.67 | 2 | 4 | 6 | 0 | 0 | -2 | 10 | Accepted |
| 3 | Gallic acid | 170.12 | 0.7 | -2.48 | -1.52 | 100.82 | 1 | 7 | 0.13 | 4 | 5 | 9 | 1 | 6 | -1 | 12 | Accepted |
| 4 | 2,3-Dihydroxybenzoic acid isomer | 154.12 | 1.2 | -1.84 | -1.75 | 80.59 | 1 | 7 | 0.13 | 3 | 4 | 7 | 1 | 6 | -1 | 11 | Accepted |
| 5 | Vanillin isomer | 152.15 | 1.21 | 1.08 | -1.65 | 46.53 | 2 | 7 | 0.22 | 1 | 3 | 4 | 1 | 6 | 0 | 11 | Accepted |
| 6 | Pantothenic acid isomer | 219.23 | -1.08 | -4.28 | -0.12 | 109.69 | 6 | 3 | 0.67 | 4 | 6 | 10 | 0 | 0 | -1 | 15 | Accepted |
| 7 | Syringic acid glucoside | 360.31 | -1.02 | -4.48 | -1.13 | 157.97 | 6 | 13 | 0.32 | 5 | 10 | 15 | 2 | 6 | -1 | 25 | Accepted |
| 8 | Neochlorogenic acid | 354.31 | -0.42 | -3.69 | -1.55 | 167.58 | 5 | 15 | 0.25 | 6 | 9 | 15 | 2 | 6 | -1 | 25 | Accepted |
| 9 | methyl gallate | 184.15 | 0.86 | 0.99 | -1.6 | 86.99 | 2 | 7 | 0.22 | 3 | 5 | 8 | 1 | 6 | 0 | 13 | Accepted |
| 10 | Catechin | 290.27 | 0.51 | 1.78 | -2.15 | 110.38 | 1 | 17 | 0.06 | 5 | 6 | 11 | 2 | 10 | 0 | 21 | Accepted |
| 11 | Esculetin isomer | 178.14 | 1.22 | 1.06 | -2.1 | 70.67 | 0 | 12 | 0 | 2 | 4 | 6 | 1 | 10 | 0 | 13 | Accepted |
| 12 | Chlorogenic acid | 354.31 | -0.42 | -3.69 | -1.55 | 167.58 | 5 | 15 | 0.25 | 6 | 9 | 15 | 2 | 6 | -1 | 25 | Accepted |
| 13 | Caffeic acid | 180.16 | 1.15 | -1.8 | -1.76 | 80.59 | 2 | 8 | 0.2 | 3 | 4 | 7 | 1 | 6 | -1 | 13 | Accepted |
| 14 | Vanillic acid glucoside | 330.29 | -1.51 | -4.2 | -0.71 | 148.74 | 5 | 13 | 0.28 | 5 | 9 | 14 | 2 | 6 | -1 | 23 | Accepted |
| 15 | Dihydromyricetin isomer | 320.25 | 0.59 | 1.36 | -2.38 | 147.68 | 1 | 18 | 0.05 | 6 | 8 | 14 | 2 | 10 | 0 | 23 | Accepted |
| 16 | Taxifolin isomer | 304.25 | 0.95 | 1.67 | -2.52 | 127.45 | 1 | 18 | 0.05 | 5 | 7 | 12 | 2 | 10 | 0 | 22 | Accepted |
| 17 | Dihydrophaseic acid | 282.33 | 0.89 | -2.25 | -1.95 | 89.82 | 3 | 12 | 0.2 | 3 | 5 | 8 | 1 | 9 | -1 | 20 | Accepted |
| 18 | (2S)-5,7,2’,6’-Tetrahydroxyflavanone isomer | 288.25 | 2.16 | 2.4 | -3.19 | 107.22 | 1 | 18 | 0.05 | 4 | 6 | 10 | 2 | 10 | 0 | 21 | Accepted |
| 19 | Naringenin 7-O-glucoside isomer | 434.39 | 0.65 | 0.56 | -2.85 | 166.14 | 4 | 24 | 0.14 | 6 | 10 | 16 | 3 | 10 | 0 | 31 | Accepted |
| 20 | Vanillic acid isomer | 168.15 | 1.43 | -1.89 | -1.87 | 69.59 | 2 | 7 | 0.22 | 2 | 4 | 6 | 1 | 6 | -1 | 12 | Accepted |
| 21 | Naringenin 6-C-glucoside isomer | 434.39 | -0.07 | -0.18 | -2.46 | 177.14 | 3 | 24 | 0.11 | 7 | 10 | 17 | 3 | 10 | 0 | 31 | Accepted |
| 22 | Ferulic acid | 194.18 | 1.51 | -1.6 | -1.98 | 69.59 | 3 | 8 | 0.27 | 2 | 4 | 6 | 1 | 6 | -1 | 14 | Accepted |
| 23 | p-Hydroxybenzoic acid | 138.12 | 1.58 | -1.58 | -1.9 | 60.36 | 1 | 7 | 0.13 | 2 | 3 | 5 | 1 | 6 | -1 | 10 | Accepted |
| 24 | Ferulic acid isomer | 194.18 | 1.51 | -1.6 | -1.98 | 69.59 | 3 | 8 | 0.27 | 2 | 4 | 6 | 1 | 6 | -1 | 14 | Accepted |
| 25 | Phlorizin isomer | 436.41 | 0.54 | 0.86 | -2.59 | 177.14 | 7 | 19 | 0.27 | 7 | 10 | 17 | 3 | 6 | 0 | 31 | Accepted |
| 26 | azelaic acid | 188.22 | 1.57 | -3.65 | -1.47 | 80.26 | 8 | 2 | 0.8 | 2 | 4 | 6 | 0 | 0 | -2 | 13 | Accepted |
| 27 | Phlorizin | 436.41 | 0.54 | 0.86 | -2.59 | 177.14 | 7 | 19 | 0.27 | 7 | 10 | 17 | 3 | 6 | 0 | 31 | Accepted |
| 28 | Abscisic acid isomer | 264.32 | 1.61 | -0.51 | -2.3 | 77.43 | 3 | 10 | 0.23 | 2 | 4 | 6 | 1 | 6 | -1 | 19 | Accepted |
| 29 | Myricetin | 318.24 | 1.18 | 0.63 | -2.85 | 151.26 | 1 | 18 | 0.05 | 6 | 8 | 14 | 2 | 10 | 0 | 23 | Accepted |
| 30 | Kaempferol-7-O-rhamnoside | 432.38 | 0.59 | 0.96 | -2.93 | 170.05 | 3 | 24 | 0.11 | 6 | 10 | 16 | 3 | 10 | 0 | 31 | Accepted |
| 31 | Trilobatin | 436.41 | 0.54 | 1.62 | -2.59 | 177.14 | 7 | 19 | 0.27 | 7 | 10 | 17 | 3 | 6 | 0 | 31 | Accepted |
| 32 | Myricetin isomer | 318.24 | 1.18 | 0.63 | -2.85 | 151.26 | 1 | 18 | 0.05 | 6 | 8 | 14 | 2 | 10 | 0 | 23 | Accepted |
| 33 | Eriodictyol | 288.25 | 2.02 | 2.41 | -3.1 | 107.22 | 1 | 18 | 0.05 | 4 | 6 | 10 | 2 | 10 | 0 | 21 | Accepted |
| 34 | Quercetin | 302.24 | 1.54 | 1.01 | -2.99 | 131.03 | 1 | 18 | 0.05 | 5 | 7 | 12 | 2 | 10 | 0 | 22 | Accepted |
| 35 | Luteolin | 286.24 | 2.53 | 1.56 | -3.52 | 110.8 | 1 | 18 | 0.05 | 4 | 6 | 10 | 2 | 10 | 0 | 21 | Accepted |
| 36 | Isorhamnetin isomer | 316.26 | 1.87 | 1.19 | -3.19 | 120.03 | 2 | 18 | 0.1 | 4 | 7 | 11 | 2 | 10 | 0 | 23 | Accepted |
| 37 | Naringenin | 272.25 | 2.52 | 2.72 | -3.33 | 86.99 | 1 | 18 | 0.05 | 3 | 5 | 8 | 2 | 10 | 0 | 20 | Accepted |
| 38 | Phloretin | 274.27 | 2.63 | 3.8 | -3.19 | 97.99 | 4 | 13 | 0.24 | 4 | 5 | 9 | 2 | 6 | 0 | 20 | Accepted |
| 39 | Kaempferol | 286.24 | 1.9 | 1.35 | -3.13 | 110.8 | 1 | 18 | 0.05 | 4 | 6 | 10 | 2 | 10 | 0 | 21 | Accepted |
| 40 | Chrysoeriol | 300.26 | 3.1 | 1.72 | -3.87 | 99.8 | 2 | 18 | 0.1 | 3 | 6 | 9 | 2 | 10 | 0 | 22 | Accepted |
